# Late-life depression accentuates cognitive weaknesses in older adults with small vessel disease

**DOI:** 10.1038/s41386-021-00973-z

**Published:** 2021-02-09

**Authors:** Lauren E. Oberlin, Matteo Respino, Lindsay Victoria, Lila Abreu, Matthew J. Hoptman, George S. Alexopoulos, Faith M. Gunning

**Affiliations:** 1 Department of Psychiatry, Weill Cornell Medicine, New York, NY USA; 2 Weill Cornell Institute of Geriatric Psychiatry, White Plains, NY USA; 3 Rush University Medical Center, Chicago, IL USA; 4 Clinical Research, Nathan Kline Institute, Orangeburg, NY USA; 5 Department of Psychiatry, NYU School of Medicine, New York, NY USA

**Keywords:** Predictive markers, Cognitive ageing, Ageing, Depression

## Abstract

Neuroimaging features of small vessel disease (SVD) are highly prevalent in older adulthood and associated with significant variability in clinical symptoms, yet the factors predicting these symptom disparities are poorly understood. We employed a novel metric of SVD, peak width of skeletonized mean diffusivity (PSMD), to elucidate the relationship of late-life depression (LLD) to the cognitive presentation of vascular pathology. A total of 109 older adults without a diagnosis of a neurocognitive disorder were enrolled in the study; 44 with major depressive disorder and 65 age-matched controls. Subjects completed neuropsychological testing and magnetic resonance imaging including FLAIR and diffusion tensor imaging sequences, from which white matter hyperintensity volume and diffusion metrics (fractional anisotropy, mean diffusivity, PSMD) were quantified. In hierarchical models, the relationship between vascular burden and cognitive performance varied as a function of diagnostic status, such that the negative association between PSMD and processing speed was significantly stronger in participants with LLD compared to controls. Greater PSMD also predicted poorer performance on delayed memory and executive function tasks specifically among those with LLD, while there were no associations between PSMD and task performance among controls. PSMD outperformed conventional SVD and diffusion markers in predicting cognitive performance and dysexecutive behaviors in participants with LLD. These data suggest that LLD may confer a vulnerability to the cognitive manifestations of white matter abnormalities in older adulthood. PSMD, a novel biomarker of diffuse microstructural changes in SVD, may be a more sensitive marker of subtle cognitive deficits stemming from vascular pathology in LLD.

## Introduction

Cerebral small vessel disease (SVD) refers to a set of pathological processes impacting the small perforating arterioles, capillaries, and venules. Neuroradiologic evidence of SVD is present to some extent in most individuals aged 60 years and over [1–3] and is a significant contributor to cognitive deficits, gait disturbances, stroke, and progression to dementia [2, 4, 5]. Cognitive deficits frequently associated with SVD include reduced processing speed and executive dysfunction [1, 6–8]. However, the pattern and extent of cognitive deficits is highly variable [1, 7], even among those with neuroradiologically similar degrees of vascular disease burden [7, 9, 10]. Individual differences in the cognitive manifestation and course of SVD suggest that other factors may compromise the capacity to preserve cognitive abilities in the setting of white matter abnormalities [2, 3, 9]. A better understanding of the factors that contribute to these symptom disparities would inform pathophysiologic mechanisms underlying specific cognitive phenotypes in SVD.

Late-life depression (LLD) may influence the presence and extent of cognitive deficits among older adults with SVD, creating a vulnerability that contributes to the clinical expression of cerebrovascular pathology. Cognitive deficits are common in LLD, and often involve impairments in processing speed, episodic memory, and executive functioning [11–15]. Though most prominent during a depressive episode, these deficits frequently persist following antidepressant treatment [15–18]. Sustained cognitive deficits in individuals suffering from LLD are associated with accelerated rates of cognitive and functional decline, disability, and progression to dementia [19–23]. The mechanisms linking severity of depression to cognitive dysfunction are not well understood. However, abnormalities in hormonal regulation, immune signaling, neurotrophic support, and lipid metabolism [24, 25] may contribute to cognitive deficits in the presence of cerebrovascular burden, which is common in LLD [26–28].

Fundamental questions remain regarding the relationship of depression to the cognitive manifestations of SVD, particularly in samples without overt neurocognitive syndromes. One hypothesis is that the neurobiological processes that predispose individuals to LLD may exacerbate cognitive vulnerabilities in the presence of SVD. Thus, LLD may contribute to the cognitive heterogeneity associated with white matter abnormalities in older adulthood, exacerbating processing speed deficits and accentuating impairments in higher-order domains. Studies evaluating this hypothesis have largely relied on macrostructural indices of SVD, including white matter hyperintensities (WMH) and lacunes of presumed vascular origin [26, 29], with mixed support [25–28]. However, SVD is a diffuse brain disorder exerting microstructural damage well beyond visible lesions [2, 3].

Advanced quantitative techniques involving diffusion-weighted imaging, which detect subtle tissue alterations in normal-appearing white matter [30], may surpass traditional indices in capturing the clinical extent and pathophysiology of SVD. A novel diffusion-weighted imaging-derived metric, peak width of skeletonized mean diffusivity (PSMD), has been validated as a marker for SVD and shows robust associations with cognitive deficits related to vascular pathology [30]. By combining skeletonization and histogram analysis, PSMD captures the distribution of mean diffusivity (MD) values across the white matter compartment, providing a global estimate of diffuse white matter dysfunction [30]. In several recent studies, PSMD outperformed conventional MRI measures in detecting cognitive deficits in samples with sporadic and inherited SVD [30–32], and correlated more strongly with cognitive performance in healthy elderly community populations with imaging features of SVD (WMH) [33–36]. PSMD has not yet been evaluated in depressed older adults but may enable more precise characterization of the scope of cognitive deficits associated with vascular pathology in LLD.

This study sought to assess the relationship of depression to the cognitive presentation of vascular pathology, quantified using PSMD, in a sample of depressed older adults and control subjects without a diagnosis of a neurocognitive disorder. We predicted that greater PSMD would be associated with reduced processing speed across diagnostic groups, and tested the hypothesis that cognitive deficits would be amplified and extend to higher order domains with increased vascular burden among those with LLD. Further, we examined the clinical utility of PSMD in LLD by evaluating the predictive precision of PSMD relative to traditional indices of diffusion and vascular disease burden. We hypothesized that, relative to conventional MR and diffusion metrics, PSMD would be more strongly associated with cognitive deficits.

## Materials and methods

### Participants

The study included participants with LLD and a nonpsychiatric comparison group, aged 60–85 years. Participants with LLD met DSM-IV criteria for Major Depressive Disorder (MDD) and were required to score 18 or greater on the 24-item Hamilton Depression Rating Scale (HAM-D). Depressed participants were recruited to participate in an escitalopram treatment trial, and had no history of psychosis or manic or hypomanic episodes. Participants in the non-psychiatric comparison group had no history or presence of any psychiatric disorder. Participants were recruited through flyers, advertisements, or referrals from healthcare providers. All participants provided written informed consent approved by the Weill Cornell Medical College and Nathan Kline Institute Institutional Review Boards. Exclusion criteria were: presence or history of any Axis I psychiatric disorder other than MDD or co-morbid generalized anxiety disorder; high suicide risk; history of electroconvulsive therapy; ongoing treatment with drugs associated with depression (e.g. steroids, alpha-methyl-dopa, clonidine, reserpine, tamoxifen, or cimetidine); acute or severe medical illness; mild cognitive impairment (MCI) or dementia by DSM-IV criteria; history or presence of neurological disease; and MRI contraindications. Further details can be found in previously published work, which relied on overlapping samples but involved different MRI sequences and/or analyses [28, 37].

### Diagnostic assessments

Participants with LLD underwent a comprehensive evaluation by a study clinician. DSM-IV diagnosis was assigned based on the Structured Clinical Interview for DSM-Revised, and study inclusion required a HAM-D score ≥18. Participants were not on antidepressants at the time of study procedures. Those who were previously prescribed an antidepressant underwent a 2-week washout procedure under the care of a study psychiatrist. Depression severity was rated during a comprehensive baseline assessment using the Montgomery–Åsberg Depression Rating Scale (MADRS). All participants completed a brief vascular risk factor questionnaire [38], comprised of binary items regarding presence or absence of diabetes mellitus, smoking, heart failure, intermittent claudication, atrial fibrillation, left ventricular hypertrophy, and anti-hypertensive medication use. MCI was assessed using the criteria outlined by Petersen [39]. If these criteria were met, participants underwent a comprehensive neuropsychological battery [40] to facilitate classification and exclusion of MCI. In a consensus conference, clinician investigators specializing in geriatric psychiatry and neuropsychology ruled out MCI based on review of neuropsychological tests, history, and overall function.

#### Neuropsychological assessment

Participants completed a neuropsychological assessment including measures of global functioning (Dementia Rating Scale), processing speed (Trail Making Test (TMT), Part A), immediate and delayed verbal memory (Hopkins Verbal Learning Test-Revised (HVLT)), and executive function (semantic fluency (Animal Naming), Stroop Interference [41, 42], TMT, Part B-A). Dysexecutive behavior was assessed using the Executive Function subscale of the Frontal Systems Behavior Scale (FrSBe). In this study, the term cognitive weaknesses refers to relative cognitive difficulties in our sample of older adults without a diagnosis of or frank neurocognitive disorder.

### MRI data acquisition

MRI data were acquired on a 3T Siemens Tim Trio equipped with a 32-channel head coil at the Nathan Kline Institute for Psychiatric Research. Anatomical imaging included a turbo dual echo scan, with high-resolution whole brain images acquired using a 3D T1-weighted MPRAGE and T2-weighted FLAIR. The acquisition parameters were as follows: MPRAGE: TR = 2500 ms, TE = 3.5 ms, slice thickness = 1 mm, TI = 1200 ms, 192 axial slices, matrix = 256 × 256 (voxel size = 1 mm isovoxel), FOV = 256 mm, IPAT = 2, flip angle = 8°; FLAIR: 64 slices with TR = 9000 ms, TE = 111 ms, TI = 2500 ms, FOV = 192 × 256 mm, matrix 192 × 256, slice thickness = 2.5 mm, IPAT = 2, flip angle = 120°.

Diffusion weighted imaging data of 72 contiguous slices (parallel to the AC-PC plane) were acquired with a spin-echo EPI pulse sequence using 30 diffusion-encoding directions (*b*-value = 1000 s/mm^2^) with 6 nondiffusion weighted b0 (0 s/mm^2^) and the following parameters: TR = 9000 ms, TE = 91 ms, FOV = 256 × 256 mm, flip angle = 90°, voxel size = 2 × 2 × 2 mm, acquisition matrix size = 128 × 128.

### White matter quantification

#### WMH, whole-brain fractional anisotropy, and MD

T1 and FLAIR images were visually evaluated to exclude subjects with macroscopic artifacts. Two sets of raters (MR, MS; LO, LA) independently performed a group-blinded visual rating of WMH severity using the operational criteria of the age-related white matter changes (ARWMC) scale [43, 44]. The inter-rater agreement was measured as intraclass correlation coefficient (ICC = 0.95). The Brain Intensity Abnormality Classification Algorithm (BIANCA) toolbox implemented in FSL was used to segment WMH. BIANCA is a fully automated, supervised method for WMH detection, based on the k-nearest neighbor algorithm to create a probability map of WMH [43]. A training dataset of manually segmented WMH masks was created from 12 subjects’ FLAIR images and used by BIANCA to segment WMH. Visual ratings were used to inform the probability thresholds, with 0.8 assigned to participants with visually defined high WMH burden, 0.9 for moderate, and 0.99 for mild, based on ARWMC scores (<5, 5–9, ≥10) as previously described [28]. A mask of WMH was generated and the total volume of hyperintensities was calculated. A visual check was performed on each WMH mask for minimal manual adjustments. WMH smaller than 3 voxels were deleted [43]. Intracranial volume was obtained through a sum of gray matter, white matter, and ventricular cerebrospinal fluid [45]. A continuous measure of normalized WMH burden was calculated as the percentage of the ratio between WMH volume and intracranial volume. Three subjects had missing or incomplete FLAIR data and were excluded from select models. Tract-averaged whole-brain fractional anisotropy (FA) and MD were derived from deterministic tractography implemented in DSI Studio [46], and served as covariates in select statistical models.

#### Peak-width of skeletonized mean diffusivity

PSMD was calculated using a publicly available shell script (www.psmd-marker.com). Computation involved skeletonization of the FA volume of each individual (implemented in FSL v5.0–tract-based spatial statistics), using the FMRIB 1 mm FA template and applying a threshold of 0.2 to the FA map [47]. MD volumes were then projected onto the mean FA skeleton using the FA-derived projection parameters. To reduce CSF partial volume contamination, MD skeletons were further masked with a standard skeleton thresholded at an FA value of 0.3 and a custom mask provided with the PSMD toolbox to exclude regions adjacent to the ventricles [30]. Finally, using a histogram analysis, PSMD was calculated as the difference between the 95th and 5th percentiles of the voxel-based MD values within each subject’s MD skeleton.

### Statistical analysis

Sample characteristics were assessed continuously and categorically by diagnostic status using two-sample *t*-tests, chi-squared tests, and Mann–Whitney *U* Tests. Normality of continuous data was tested using the Shapiro–Wilk test. Multivariate linear regression was used to evaluate the association between PSMD and performance on measures of processing speed (TMT, Part A), memory (HVLT immediate, delayed recall), and executive function (Stroop Interference, TMT B-A, Animal Naming). Diagnostic status was included as a dichotomous predictor and moderator to assess whether the relationship between PSMD and cognitive performance was greater in magnitude among participants with LLD. Thus, hierarchical models included PSMD and diagnostic status as independent predictors, and their interaction product [48]. All models adjusted for age, sex, and years of education. Significant terms in demographically adjusted models were subsequently evaluated in conservatively adjusted models controlling for normalized WMH volume and mean whole-brain FA.

To decompose significant interaction terms, we assessed the conditional relationship of the focal predictor at the two values of the moderator [48]. Those that differed from zero were subjected to secondary within-group analysis involving additional adjustment for WMH volume and mean FA. This enabled us to evaluate whether PSMD provided additional predictive value in detecting cognitive weaknesses in LLD beyond traditional measures of SVD and diffusion parameters. We employed a hierarchical linear regression model, in which demographics were entered first, followed by normalized WMH volume and mean FA, and finally by PSMD. Multicollinearity between predictors can confound estimates of individual predictor weights. Given associations between white matter indices, we tested for collinearity via examination of zero-order correlations, tolerance, and the variance inflation factor. Global mean MD was included as a predictor in select sensitivity models to further probe the discriminative value of PSMD. The Akaike Information Criterion was used to evaluate model fit. Significance was set at *p* < 0.05. Analyses were run in SPSS (V25) and Jamovi by R [49, 50].

## Results

### Descriptive statistics

Demographic and clinical characteristics of the sample are summarized in Table 1. Relative to controls (*n* = 65), those with LLD (*n* = 44) had fewer years of education and greater MADRS scores. Groups did not differ with respect to WMH volume, mean FA, or PSMD. PSMD was positively correlated with WMH (*r* = 0.62; *p* < 0.001) and mean MD (*r* = 0.76; *p* < 0.001) and negatively correlated with mean FA (*r* = −0.6; *p* < 0.001). Data on cognitive task performance can be found in Supplementary Table 1.

**Table 1:** Demographic, clinical, and imaging characteristics.

	Total sample	Controls	LLD	Statistics
	*n* = 109	*n* = 65	*n* = 44	
	*M*, SD or % (*N*)	*M*, SD or % (*N*)	*M*, SD or % (*N*)	(*t*, *χ*^2^)
Age, y	72.07 ± 6.5	72.38 ± 6.3	71.61 ± 7.01	t = 0.602
Gender (Female)	62.4% (68)	60% (39)	65.9% (29)	*χ*^2^ = 0.39
Education, y	16 ± 2.7	16.6 ± 2.28	15.1 ± 3	*t* = 2.9*
HAM-D (24 item)	10.35 ± 11.4	1.28 ± 1.3	23.75 ± 4.1	*t* = −35.2*
MADRS	11.03 ± 12.52	1.1 ± 1.4	25.75 ± 4.4	*t* = −36.5*
Dementia Rating Scale	139.43 ± 4.8	139.75 ± 4.9	138.95 ± 4.6	*t* = 0.40
Vascular Risk Measure	0.82 ± 0.93	0.71 ± 0.82	1.0 ± 1.06	*t* = −1.6
Normalized WMH volume (% ICV)	0.19 ± 0.28	0.15 ± 0.23	0.27 ± 0.33	*t* = −1.68
Mean FA	0.37 ± 0.02	0.37 ± 0.02	0.37 ± 0.02	*t* = 1.23
PSMD 10^−4^ mm^2^/s	3.52 ± 0.67	3.4 ± 0.55	3.64 ± 0.83	*t* = −1.29

Vascular Risk Measure refers to a self-reported clinical measure of vascular illness.

*HAM-D* Hamilton Depression Rating Scale, *FA* fractional anisotropy, *ICV* intracranial volume, *MADRS* Montgomery–Asberg Depression Rating Scale, *PSMD* peak width of skeletonized mean diffusivity, *WMH* white matter hyperintensities

*p  <  0.05.

### Associations among white matter abnormalities, mood, and cognition

Relative to controls, participants with LLD performed more poorly on measures of immediate memory, delayed memory, attentional set-shifting, and semantic fluency, while performance on measures of processing speed and cognitive inhibition did not differ between groups (Table 2). Collapsed across groups, PSMD was associated with reduced processing speed and poorer semantic fluency in demographically adjusted models.

**Table 2:** Parameter estimates from hierarchical linear regression models of diagnostic status, PSMD, and their interaction product as predictors of cognitive performance.

	PSMD (10^−4^ mm^2^/s)			Group (LLD vs. controls)			PSMD × Group			
Cognitive measure	Estimate (df)	SE	*p*-Value	Estimate	SE	*p*-Value	Estimate	SE	*t*-Value	*p*-Value
HVLT Immediate	−0.45 (104)	1.37	0.746	−0.69	0.19	<0.001	−1.45	2.32	−0.63	0.532
HVLT Delayed	−1.64 (105)	1.36	0.232	−0.65	0.19	0.001	−5.87	2.23	−2.6	0.010
Trail Making Test, Part A	5.34 (105)	1.36	<0.001	0.27	0.19	0.157	5.17	2.26	2.29	0.024
Trails B-A	0.15 (97)	1.47	0.918	0.60	0.21	0.005	1.4	2.53	0.553	0.582
Stroop Interference	−2.38 (108)	1.44	0.112	0.29	0.21	0.167	−5.9	2.47	−2.39	0.019
Animal Naming	−3.85 (106)	1.39	0.007	−0.39	0.19	0.047	−4.7	2.31	−2.03	0.046

*SE* standard error, *HVLT* Hopkins Verbal Learning Test, *PSMD* peak width of skeletonized mean diffusivity, *LLD* late-life depression.

**Fig. 1:**
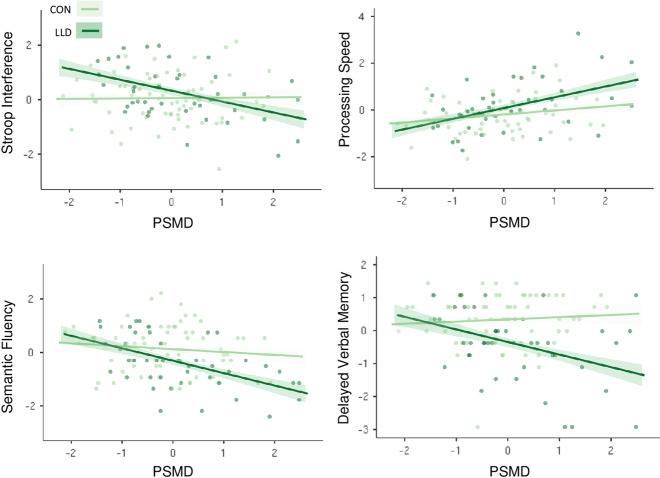
Conditional associations between PSMD (10^−4^ mm^2^/s) and cognitive performance at values of the moderator (mood status). Individual scatterplots demonstrate significant interaction effects on select cognitive measures. Controls depicted in light green and subjects with LLD depicted in dark green. The relationship between PSMD and slowed processing speed (Trail Making Test, Part A) was magnified among those with LLD. PSMD was associated with poorer performance on measures of cognitive inhibition, semantic fluency, and delayed verbal memory in subjects with LLD, but was unrelated to task performance in these domains in controls. Trail Making Test, Part A and PSMD were log-transformed, all variables were then individually normalized by *z*-transformation for visualization. Shaded areas surrounding best-fitting lines depict standard errors. PSMD peak width of skeletonized mean diffusivity, LLD late-life depression.

The association between PSMD and delayed memory performance was moderated by diagnostic status, such that elevated levels of PSMD predicted poorer delayed memory specifically among those with LLD (Table 2). This Group × PSMD interaction was also observed for measures of processing speed, cognitive inhibition, and semantic fluency. To decompose interaction terms, we evaluated the significance of the slopes at each value of the moderator. PSMD was associated with reduced processing speed in controls and subjects with LLD, although this relationship was stronger among subjects with LLD (Fig. 1). Greater PSMD was associated with poorer performance on measures of delayed memory, cognitive inhibition, and semantic fluency in subjects with LLD, while PSMD was unrelated to performance on these tasks among controls. Interaction terms for processing speed, delayed memory, and cognitive inhibition remained significant in conservative models controlling for WMH and mean FA. The interaction term for semantic fluency was attenuated in the conservatively adjusted model.

### Associations between white matter indices and cognition in LLD

Secondary multivariate hierarchical regression models were conducted in the subset of participants with LLD (*n* = 44) to evaluate the potentially distinct contribution of PSMD to variability in cognitive performance beyond conventional white matter indices. There was no association between PSMD, mean FA, mean MD or WMH, and depression severity (MADRS). With the exception of mean MD, the variance inflation factor ranged from 1–1.5 for model predictors, suggesting collinearity among predictors was low [51]. Thus, mean MD was evaluated as a unique predictor in follow-up sensitivity models.

Consistent with interaction terms, among those with LLD, PSMD was associated with poorer performance on measures of semantic fluency, cognitive inhibition, processing speed, and delayed memory after adjustment for demographic factors and depression severity. PSMD remained significantly associated with semantic fluency and cognitive inhibition following further adjustment for WMH volume, with PSMD explaining an additional 9.1% and 20.9% of the variance above and beyond all other model predictors, respectively (Table 3). These relationships were unchanged in exploratory analyses adjusting for a clinical measure of vascular illness. The association between PSMD and semantic fluency was no longer significant (*p* = 0.066) after inclusion of mean FA as a nuisance variable, whereas the relationship with delayed memory reached significance (Supplementary Table 2).

**Table 3:** Parameter estimates from hierarchical regression models examining associations between PSMD, WMH, and executive processes in LLD.

	Semantic fluency			Cognitive inhibition			Executive dysfunction (FrSBe)		
	*β*	*t*-Value	*R*^2^	*β*	*t*-Value	*R*^2^	*β*	*t*-Value	*R*^2^
*Step 1*									
Age	−0.306*	−2.029	0.21	−0.28	−1.7	0.087	−0.23	−1.368	0.111
Gender	0.04	0.256		0.007	0.04		0.14	0.806	
Education	0.212	1.408		0.037	0.23		−0.23	−1.387	
MADRS	0.24	1.607		0.043	0.27		0.071	0.438	
*Step 2*									
WMH volume	−0.384*	−2.353	0.103	−0.05	−0.29	0.002	0.247	1.302	0.042
*Step 3*									
PSMD	−0.525*	−2.338	0.091	−0.80**	−3.27	0.209	0.50*	2.08	0.097

*R*^2^ values reflect total variance accounted for by age, gender, education, and MADRS scores in Step 1, and *R*^2^ change in Steps 2 and 3.

*FrSBe* Frontal Systems Behavior Scale, *MADRS* Montgomery–Åsberg Depression Rating Scale, *PSMD* peak width of skeletonized mean diffusivity, *WMH* white matter hyperintensities.

*p < 0.05; **p < 0.001.

**Fig. 2:**
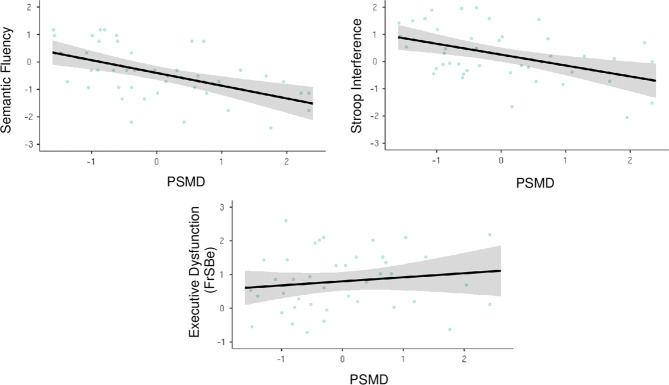
Associations between PMSD, executive function performance, and dysexecutive behavior among participants with LLD. Scatterplot of associations between PSMD (10^−4^ mm^2^/s), executive function performance, and self-reported dysexecutive behavior on the FrSBe in the depressed subsample (*n* = 44). PSMD was log-transformed, all variables were then individually normalized by *z*-transformation for visualization. Shaded areas surrounding best-fitting lines reflect 95% confidence intervals. FrSBe Frontal Systems Behavior Scale, PSMD peak width of skeletonized mean diffusivity.

Given select associations of PSMD with objective measures of executive functioning, an exploratory analysis was conducted with the FrSBe Executive Function subscale to evaluate whether PSMD was also a significant predictor of dysexecutive behavior. Hierarchical models revealed a positive association, with PSMD accounting for an additional 9.7% of the variance in self-reported dysexecutive behavior (Fig. 2). PSMD outperformed mean MD with respect to model fit on measures of dysexecutive behavior, cognitive inhibition, and delayed memory (Supplementary Table 3).

## Discussion

The principal finding of this study is that the presence of depression modifies the nature and extent of cognitive difficulties associated with cerebrovascular burden in a sample of community-dwelling older adults. Greater cerebrovascular burden, quantified using PSMD, was exclusively associated with slowed processing speed among non-depressed individuals, but predicted broader and more pronounced weaknesses in processing speed, delayed memory, and executive function among those with LLD. To our knowledge, this is the first study to use PSMD as a measure of vascular pathology in LLD. PSMD surpassed conventional SVD and diffusion markers in detecting relationships with executive performance and dysexecutive behaviors. These observations provide insight into the relationship of depression to the cognitive sequalae of white matter abnormalities in older adulthood and demonstrate the usefulness of PSMD as a sensitive and robust marker of subtle cognitive weaknesses associated with vascular pathology in LLD.

The cognitive manifestations of SVD are highly variable, both in nature and severity. While processing speed is prominently affected [6], associations within the executive domain are somewhat weaker [1, 7], particularly in cohorts with milder degrees of vascular pathology. Some studies also report SVD-related deficits in memory and language [3, 52], yet others fail to detect these relationships [6, 8]. Individual differences in the cognitive presentation of SVD have been associated with the number, location, and combination of lesion types [2, 53]. However, heterogeneity in cognitive function is observed even among those with neuroradiologically similar degrees of vascular burden [7, 9, 10]. Identifying additional factors that modulate brain resilience, or the capacity to tolerate a degree of brain pathology before clinical symptoms manifest, is a critical next step in the detection and management of these complex disease patterns [2, 3].

Our findings suggest that the ability to endure microvascular abnormalities with minimal cognitive consequences may be compromised by LLD. Depressed older adults performed more poorly on measures of immediate and delayed memory and executive function compared to controls, consistent with prior literature [12–15]. PSMD was independently associated with slowed processing speed and poorer semantic fluency across the full sample, which aligns with recent reports [1, 6, 30, 33]. In addition to these main effects, there was a synergistic relationship such that the combination of LLD and elevated vascular burden was more strongly associated with performance decrements than would be expected by their additive contributions. The association between PSMD and slowed processing speed was significantly greater among those with LLD compared to non-depressed individuals. Moreover, higher PSMD was associated with poorer delayed memory, semantic fluency, and cognitive inhibition among participants with LLD, while PSMD was unrelated to task performance among controls. This pattern suggests that non-depressed older adults may be better able to compensate for the impacts of elevated vascular burden, while those with LLD may present amplified cognitive deficits across multiple domains of functioning [54]. Given quantitively similar levels of WMH and PSMD across groups, our findings suggest that LLD may act as a “tipping point”, facilitating the expression of cognitive deficits associated with white matter abnormalities in older adulthood.

One interpretation of our findings is that biological processes occurring during depression may compound the pathogenic impact of SVD and lead to the manifestation of deficits. Changes in immune signaling, intracellular signaling, clotting processes, and lipid and protein homeostasis during LLD [24, 25] are some of the mechanisms that may exacerbate cognitive vulnerabilities in the presence of SVD. Thus, the brains of older adults with LLD may be structurally and functionally more compromised beyond just SVD, rendering those with depression more likely to express cognitive deficits. Consistent with this view, LLD is associated with alterations in functional connectivity of the default mode and cognitive control networks even after accounting for WMH [55]. Individuals with SVD and LLD also exhibit more severe disruptions in global network efficiency and lower nodal efficiency than those with SVD alone, highlighting the synergistic impact of LLD and SVD on structural brain networks [56].

An alternative interpretation of these data is that both depression and cognitive impairment originate from SVD [26, 57]. In such a case, a broader or more severe SVD would be expected in subjects with LLD, resulting in the manifestation of cognitive deficits. However, this explanation is not supported by our data as we failed to find differences in SVD markers between depressed subjects and healthy controls. Moreover, SVD markers were not associated with depression severity among those with LLD. The use of cross-sectional data limits inferences that can be drawn about the nature of the moderating relationships and future longitudinal studies are required to further decompose these relationships. However, our preliminary findings suggest that LLD may impact the presence and extent of cognitive deficits among those with evidence of mild to moderate SVD pathology.

PSMD may have several advantages over other assessment methods of SVD in LLD. In hierarchical models, PSMD accounted for 9–21% of the variance on measures of cognitive inhibition, semantic fluency, and dysexecutive behavior above and beyond all other model predictors, including WMH. PSMD was also more strongly associated with performance on select cognitive tasks compared to traditional diffusion metrics mean FA and mean MD. The relative superiority of PSMD observed here converges with recent evidence in healthy aging [33–36, 58], preclinical Alzheimer’s disease [59], and sporadic and inherited SVD [30, 31, 60] and highlights the clinical relevance of this marker in LLD.

Along with being fully automated and publicly available, PSMD is less susceptible to partial volume effects from CSF contamination compared to established DTI metrics [30]. Moreover, PSMD has been integrated into the latest generation of clinical trials for vascular cognitive impairment (see https://markvcid.partners.org/) and is found to be selectively sensitive to vascular pathology, rather than general/non-specific neurodegeneration [30, 59]. In our sample, PSMD provided significant, additional predictive value beyond conventional measures, demonstrating particularly robust associations in the executive domain. Along with visible lesions, diffuse disruptions in the microarchitecture may contribute to cognitive deficits in LLD. Assessing PSMD alongside traditional metrics may help to further characterize the impact of vascular pathology on cognition in LLD, and enhance detection of these relationships in those with mild to moderate degrees of vascular pathology.

Limitations of the study include a relatively limited neuropsychological battery. Investigation in a larger sample entailing several measures of executive function, memory, and additional processes (visuospatial, language) may allow for a more nuanced understanding of the breadth and specificity of deficits associated with LLD in SVD. Although the sample is risk-enriched for SVD given their age, participants exhibited a rather low lesion burden. Our aim was to target gaps in the literature among populations with mild to moderate degrees of white matter abnormalities. However, observed relationships may be more pronounced or vary in more severely affected individuals. In depressed participants, the anti-depressant washout may have impacted performance during the baseline cognitive assessment. There was a difference between groups in years of education. As educational attainment is associated with cognitive functioning in late-life [61], this was systematically adjusted for in all statistical models although this may not adequately account for the impact of differences in educational attainment on cognitive performance. While we attempted to screen out subjects with MCI, it is possible that some individuals may have had MCI that was not detected by our evaluation process.

Additional radiological manifestations of SVD including lacunes, microbleeds, and enlarged perivascular spaces were not assessed. While these markers tend to have weaker associations with cognition than WMH [30, 62], particularly in stroke and dementia-free populations, composite scores reflecting a combination of MR features may better reflect SVD severity than WMH alone [9, 63]. In addition, we used an estimate of global WMH, although some evidence indicates that the regional distribution of WMH may share a stronger relationship with specific measures of cognition [52, 64, 65]. While PSMD is a sensitive measure of diffuse cerebrovascular burden, the heterogenous processes that contribute to vascular damage, along with their regional specificity, cannot be distinguished with this metric. Finally, as participants in this study were in a current depressive episode, we cannot ascertain whether the relationships observed would persist in a remitted state. Future studies may use PSMD to further interrogate the associations between SVD and enduring cognitive deficits in remitted LLD.

## Conclusions

Greater SVD burden as defined by PSMD was associated with broader and more prominent weaknesses in cognitive performance among those with LLD, while healthy controls exhibited a relative cognitive resilience to elevated vascular burden. Our findings suggest that biological processes occurring during depression may add to the disruption caused by SVD and lead to the clinical expression of cognitive deficits. The specificity and scope of cognitive deficits associated with SVD in depressed older adults may be better detected by PSMD than conventional metrics, highlighting the clinical utility of this marker in LLD.

## Funding and disclosure

This work was supported by the National Institute of Mental Health (NIMH) grants R01 MH097735 (Gunning) and T32 MH019132-30 (Alexopoulos). Dr. Alexopoulos serves at the speakers’ bureaus of Takeda, Lundbeck, Otsuka, and Sunovion. The other authors have no disclosures to report.

## Supplementary information

Supplementary Table 1

Supplementary Table 2

Supplementary Table 3
