# Supplementary material for: Late-life depression accentuates cognitive weaknesses in older adults with small vessel disease
Source: Neuropsychopharmacology. 2021 Feb 9;47(2):580–7. doi: 10.1038/s41386-021-00973-z (PMC8674355; doi:10.1038/s41386-021-00973-z)
Supplement: Supplementary file 1 — Supplementary Table 1 [file 41386_2021_973_MOESM1_ESM.docx]

|  | **Controls** | |  | **LLD** | |  | **Statistics** | |
| --- | --- | --- | --- | --- | --- | --- | --- | --- |
|  | N = 65 | |  | N = 44 | |  |  |  |
|  | *M* | SD |  | *M* | SD |  | F statistic | p value |
| Semantic Fluency (animals) | 21.10 | 4.33 |  | 18.52 | 4.59 |  | 5.96 | 0.016 |
| Immediate Verbal Memory (HVLT) | 24.30 | 3.53 |  | 21.23 | 4.18 |  | 13.07 | < .001 |
| Delayed Verbal Memory (HVLT) | 8.95 | 2.11 |  | 6.89 | 2.97 |  | 13.24 | < .001 |
| Digit Span Forward | 8.42 | 2.40 |  | 7.49 | 2.56 |  | 2.09 | 0.151 |
| Digit Span Backward | 6.57 | 1.77 |  | 5.79 | 2.11 |  | 2.27 | 0.135 |
| Digit Span Total | 14.98 | 3.47 |  | 13.28 | 4.01 |  | 3.06 | 0.083 |
| Stroop Word Reading | 96.48 | 15.25 |  | 89.02 | 16.13 |  | 2.00 | 0.16 |
| Stroop Color Naming | 62.82 | 12.69 |  | 58.80 | 12.94 |  | 1.57 | 0.213 |
| Stroop Interference | -3.70 | 7.57 |  | -1.82 | 7.48 |  | 1.30 | 0.257 |
| Trail Making Test, Part A (secs) | 39.70 | 14.02 |  | 52.67 | 33.96 |  | 4.04 | 0.047 |
| Trails B - Trails A (secs) | 53.89 | 31.19 |  | 81.57 | 42.86 |  | 8.89 | 0.004 |

**Supplementary Table 1.** Performance on cognitive tasks, stratified by group.

Education-adjusted group differences in ANCOVA models. Raw mean and standard deviation values provided for all variables, Trail Making Test, Part A and Trails B – Trails A variables were log-transformed for ANCOVA models due to skewed distributions.

Abbreviations: SD = standard deviation; HVLT = Hopkins Verbal Learning Test; M = mean; PSMD = Peak Width of Skeletonized Mean Diffusivity. LLD = late-life depression.
