# Supplementary material for: Late-life depression accentuates cognitive weaknesses in older adults with small vessel disease
Source: Neuropsychopharmacology. 2021 Feb 9;47(2):580–7. doi: 10.1038/s41386-021-00973-z (PMC8674355; doi:10.1038/s41386-021-00973-z)
Supplement: Supplementary file 2 — Supplementary Table 2 [file 41386_2021_973_MOESM2_ESM.docx]

|  |  | **Semantic Fluency** | | | |  | | **Cognitive Inhibition** | | |  | **Executive Dysfunction (FrSBe)** | | |  | **Delayed Verbal Memory** | | |
| --- | --- | --- | --- | --- | --- | --- | --- | --- | --- | --- | --- | --- | --- | --- | --- | --- | --- | --- |
|  |  | β | t-value | R^2^  Change |  | | β | | t-value | R^2^  Change |  | β | t-value | R^2^  Change |  | β | t-value | R^2^  Change |
| *Step 1* |  |  |  |  | |  | |  |  |  |  |  |  |  |  |  |  |  |
|  | Age | -0.31 | -1.97 | 0.199 | |  | | -0.28 | -1.71 | 0.092 |  | -0.23 | -1.32 | 0.121 |  | -0.33* | -2.13 | 0.232 |
|  | Gender | 0.02 | 0.12 |  |  |  | | 0.05 | 0.29 |  |  | 0.13 | 0.71 |  |  | 0.40* | 2.49 |  |
|  | Education | 0.17 | 1.06 |  |  |  | | 0.09 | 0.53 |  |  | -0.25 | -1.48 |  |  | 0.29 | 1.88 |  |
|  | MADRS | 0.25 | 1.59 |  |  |  | | 0.05 | 0.29 |  |  | 0.07 | 0.44 |  |  | -0.08 | -0.55 |  |
| *Step 2* |  |  |  |  | |  | |  |  |  |  |  |  |  |  |  |  |  |
|  | WMH Volume | -0.45* | -2.46 | 0.188 | |  | | -0.10 | -0.45 | 0.012 |  | 0.16 | 0.78 | 0.106 |  | -0.33 | -1.68 | 0.065 |
|  | Mean FA | 0.19 | 1.10 |  |  |  | | 0.06 | 0.29 |  |  | -0.30 | -1.67 |  |  | -0.21 | -1.14 |  |
| *Step 3* |  |  |  |  | |  | |  |  |  |  |  |  |  |  |  |  |  |
|  | PSMD | -0.48 | -1.90 | 0.061 | |  | | -0.9* | -3.16 | 0.208 |  | 0.38 | 1.31 | 0.042 |  | -0.62* | -2.34 | 0.10 |

*p < 0.05

R^2^ values reflect total variance accounted for by age, gender, education, and MADRS scores in Step 1, total variance accounted for by WMH volume and Mean FA in Step 2, and R^2^ change in Step 3.

Abbreviations: FrSBe = Frontal Systems Behavior Scale; FA=Fractional Anisotropy; MADRS = Montgomery–Åsberg Depression Rating Scale; PSMD = Peak Width of Skeletonized Mean Diffusivity; WMH = White matter hyperintensities

**Supplementary Table 2.** Parameter estimates from hierarchical regression models examining associations between PSMD, WMH, mean FA and cognition in LLD.
