# Supplementary material for: Late-life depression accentuates cognitive weaknesses in older adults with small vessel disease
Source: Neuropsychopharmacology. 2021 Feb 9;47(2):580–7. doi: 10.1038/s41386-021-00973-z (PMC8674355; doi:10.1038/s41386-021-00973-z)
Supplement: Supplementary file 3 — Supplementary Table 3 [file 41386_2021_973_MOESM3_ESM.docx]

**Supplementary Table 3.** Fit indices from hierarchical regression models examining associations between PSMD, mean MD and cognitive performance in LLD.

|  | **Semantic Fluency** | | | |
| --- | --- | --- | --- | --- |
|  | AIC | F | R^2^ Change | p value |
| mean MD | -12.12 | 7.2 | 0.11 | 0.011 |
| PSMD | -8.3 | 3.625 | 0.061 | 0.066 |
|  | **Cognitive Inhibition (Stroop Interference)** | | | |
|  | AIC | F | R^2^ Change | p value |
| mean MD | 8.32 | 0.874 | 0.023 | 0.357 |
| PSMD | -1.454 | 9.99 | 0.208 | 0.003 |
|  | **Delayed Verbal Memory** | | | |
|  | AIC | F | R^2^ Change | p value |
| mean MD | 6.72 | 0.382 | 0.008 | 0.541 |
| PSMD | 0.92 | 5.454 | 0.10 | 0.026 |
|  | **Processing Speed (Trails, Part A)** | | | |
|  | AIC | F | R^2^ Change | p value |
| mean MD | -2.86 | 0.261 | 0.003 | 0.613 |
| PSMD | -2.64 | 0.085 | 0.001 | 0.772 |
|  | **Executive Dysfunction (FrSBe)** | | | |
|  | AIC | F | R^2^ Change | P value |
| mean MD | 186.05 | 0.397 | 0.01 | 0.533 |
| PSMD | 184.4 | 1.72 | 0.042 | 0.199 |

*p < 0.05;

Due to collinearity between mean MD and PSMD, predictors were included in separate models to evaluate model fit.

R^2^ change values reflect change in variance explained after including PSMD or mean MD in hierarchical models adjusting for age, gender, education, MADRS, WMH volume and mean FA. F=F change statistic.

Abbreviations: AIC = Akaike Information Criterion; FrSBe = Frontal Systems Behavior Scale; MADRS = Montgomery–Åsberg Depression Rating Scale; MD = mean diffusivity; PSMD = Peak Width of Skeletonized Mean Diffusivity;WMH = White matter hyperintensities
